# Separate hydrolysis and co-fermentation for improved xylose utilization in integrated ethanol production from wheat meal and wheat straw

**DOI:** 10.1186/1754-6834-5-12

**Published:** 2012-03-12

**Authors:** Borbála Erdei, Balázs Frankó, Mats Galbe, Guido Zacchi

**Affiliations:** 1 Lund University, Department of Chemical Engineering, PO Box 124, SE-221 00 Lund, Sweden; 2 Budapest University of Technology and Economics, Department of Applied Biotechnology and Food Science, Szt Gellért tér 4, 1111 Budapest, Hungary

**Keywords:** SHF, SHCF, integration, co-fermentation, wheat hydrolysate, *Saccharomyces cerevisiae *TMB3400

## Abstract

**Background:**

The commercialization of second-generation bioethanol has not been realized due to several factors, including poor biomass utilization and high production cost. It is generally accepted that the most important parameters in reducing the production cost are the ethanol yield and the ethanol concentration in the fermentation broth. Agricultural residues contain large amounts of hemicellulose, and the utilization of xylose is thus a plausible way to improve the concentration and yield of ethanol during fermentation. Most naturally occurring ethanol-fermenting microorganisms do not utilize xylose, but a genetically modified yeast strain, TMB3400, has the ability to co-ferment glucose and xylose. However, the xylose uptake rate is only enhanced when the glucose concentration is low.

**Results:**

Separate hydrolysis and co-fermentation of steam-pretreated wheat straw (SPWS) combined with wheat-starch hydrolysate feed was performed in two separate processes. The average yield of ethanol and the xylose consumption reached 86% and 69%, respectively, when the hydrolysate of the enzymatically hydrolyzed (18.5% WIS) unwashed SPWS solid fraction and wheat-starch hydrolysate were fed to the fermentor after 1 h of fermentation of the SPWS liquid fraction. In the other configuration, fermentation of the SPWS hydrolysate (7.0% WIS), resulted in an average ethanol yield of 93% from fermentation based on glucose and xylose and complete xylose consumption when wheat-starch hydrolysate was included in the feed. Increased initial cell density in the fermentation (from 5 to 20 g/L) did not increase the ethanol yield, but improved and accelerated xylose consumption in both cases.

**Conclusions:**

Higher ethanol yield has been achieved in co-fermentation of xylose and glucose in SPWS hydrolysate when wheat-starch hydrolysate was used as feed, then in co-fermentation of the liquid fraction of SPWS fed with the mixed hydrolysates. Integration of first-generation and second-generation processes also increases the ethanol concentration, resulting in a reduction in the cost of the distillation step, thus improving the process economics.

## Background

The use of bioethanol is beneficial for several reasons. One is that it can be easily integrated into the current fuel distribution system, and another is that it will reduce the production of greenhouse gases as the raw material used is biomass. Bioethanol can be produced from lignocellulosic biomass in three main steps: pretreatment, enzymatic hydrolysis, and fermentation. Enzymatic hydrolysis and fermentation can be carried out either separately, using separate hydrolysis and fermentation (SHF), or simultaneously, using simultaneous saccharification and fermentation (SSF).

Over the past decade SSF has become the preferred process, since end-product inhibition of the enzymes can be avoided by performing fermentation in the same vessel at the same time as hydrolysis [1]. The capital investment cost of the plant is also reduced as fewer tanks are required [2]. However, a disadvantage of SSF is that the operating temperature must be a compromise between the optimal temperatures for hydrolysis and fermentation, whereas these can be optimized independently in SHF. Furthermore, the yeast produced during the SHF process can be recycled after fermentation of the hydrolysate, which is not possible in SSF. The yeast thus represents a yield loss as it is difficult to separate it from the solid residue (lignin) [3].

Wheat straw and other agricultural residues generally consist of high amounts of hemicelluloses [4]. Xylose fermentation is therefore very important for these lignocellulosic raw materials in order to effectively convert all the sugars into ethanol, to increase the concentration in the fermentation broth. However, wild-type *Saccharomyces cerevisiae*, which is the most commonly used yeast in ethanol fermentation due to its attractive properties, such as high yield of ethanol, high specific rate of fermentation [5], and its high tolerance to the end product, ethanol [6], is not able to metabolize xylose. Therefore, naturally xylose-fermenting yeasts, such as *Candida shehatae *or *Pichia stipitis *[7,8] have been widely studied since their ability to ferment xylose was discovered the early 1980s. Unfortunately, their tolerance to inhibitors [9] and ethanol [10] is limited, and they also require a very low and well-controlled supply of oxygen for effective xylose-fermentation [11,12], which makes them difficult to use in large-scale production.

The ideal solution, combining the robustness and tolerance of *S. cerevisiae *with the ability to ferment xylose has been sought by metabolic engineering. In principle, genes from bacteria and fungi encoding xylose isomerase (XI) [13,14], or genes from fungi encoding xylose reductase (XR) and xylitol dehydrogenase (XDH) [15] can be introduced into *S. cerevisiae*. The endogenous gene *XKS1 *encoding xylulokinase (XK) must also be overexpressed for the strain to be able to utilize xylose for growth and ethanol production [16]. TMB3400 [17] is an industrial strain of *S. cerevisiae *containing genes that encode for XR/XDH/XK, which is able to co-ferment xylose and glucose in non-detoxified lignocellulose hydrolysate of spruce [18,19], as well as various pretreated raw materials [20-23] in simultaneous saccharification and co-fermentation (SSCF).

In *S. cerevisiae *TMB3400, xylose and glucose are competitively transported by the same transport protein [24,25], but xylose has an approximately 200-fold lower affinity [26]. To avoid the inhibition of xylose uptake by glucose, the glucose concentration in the medium must be low. It has been reported that low, but non-zero, concentration of glucose enhances xylose utilization [25], which means that a slow release or feeding of glucose is required. This is one of the reasons why SSCF has become an interesting process option, as glucose is released during hydrolysis.

Co-fermentation of glucose and xylose in wheat straw hydrolysate has been investigated by Olofsson *et al. *[21]. Their results showed that almost complete xylose fermentation can be obtained if a controlled glucose feed is applied during fermentation. However, during SSF the release of sugar is not controlled, as all the cellulase enzymes are added at once. Thus prefermentation [20] and enzyme feeding strategies combined with fed-batch fermentation have recently been studied [27] as a means of controlled glucose release. The best result so far, 80% xylose uptake, has been achieved by Olofsson *et al. *using a yeast concentration of 4 g/L and controlled feeding of cellulases giving a glucose release rate of 2 g/L h [28]. Simultaneous glucose and xylose uptake has been modelled in a study by Bertilsson *et al*., indicating that a glucose feed rate between 5 and 10 g/L h would be suitable to obtain maximum xylose uptake rate, with a yeast cell concentration of 5 g/L [29]. The effect of controlled glucose feeding has also been studied on barley straw hydrolysate by Linde *et al. *[30], however, only 74% and 51% of the xylose was consumed by the yeast TMB3400 at glucose feed rates of 0.21 g/L h and 0.45 g/L h, respectively.

Wheat hydrolysate is derived from wheat meal, following the first generation ethanol production process through liquefaction and saccharification. The hydrolysate obtained is a glucose-rich solution as well as a potential source of nutrients, which has several advantages in the process. The use of wheat-starch hydrolysate, as a complex nutrient source, has been shown to be a potential supplement for lignocellulosic hydrolysates [31]. It also has a positive effect on glucose fermentation in SSF, increasing both the ethanol yield and concentration [32], which is important for the process economy [33]. Therefore including wheat-starch hydrolysate in the feed in SHF, which is a first-generation glucose source, provides considerable possibilities for improvement of xylose uptake and hence increase the ethanol yield and concentration.

To the best of our knowledge, no study has yet been performed to evaluate separate hydrolysis and co-fermentation (SHCF) of steam-pretreated wheat straw combined with wheat-starch hydrolysate feed as a means of improving the co-fermentation of glucose and xylose. In the present work, two process configurations for SHCF were investigated using steam-pretreated wheat straw combined with wheat-starch hydrolysate feed, employing the xylose-fermenting yeast TMB3400.

## Results and discussion

### Enzymatic hydrolysis

Enzymatic hydrolysis of the whole SPWS slurry was performed at 7.5% WIS in Config. 1 to supply fermentation with hydrolysate. Enzymatic hydrolysis was first performed using SPWS 1 material supplemented with Cellic CTec and Cellic Htec enzyme preparations at a cellulase loading of 20 FPU/g glucan. The final glucose and xylose concentrations of 55.6 g/L and 30.6 g/L, respectively, were reached after 48 h, corresponding to yields of 96% and 91% of the theoretical, respectively. Since the hemicellulose sugars are mostly released during steam pretreatment, the xylose concentration only increased slightly during hydrolysis. Enzymatic hydrolysis of wheat straw has been investigated by Jorgenssen *et al. *[34], who obtained lower cellulose and xylose conversion yield at similar WIS loading, 75% and 65%, respectively, probably due to the different pretreatment conditions (steam pretreatment at 195°C or 205°C for 12 min with no chemical addition) and a lower enzyme mixture loading (13.3 FPU/g glucan) of a less effective enzyme cocktail (Celluclast 1.5 FG L and Novozym 188).

Enzymatic hydrolysis was then performed on the SPWS 2 material with a lower enzyme loading of Cellic Ctec2 (14 FPU/g glucan) when using wheat-starch hydrolysate feed. This experiment resulted in lower sugar concentrations due to the slightly lower WIS content (7.0%), and lower yields. The glucose and xylose concentrations reached their maxima of 32.3 g/L and 15.6 g/L, respectively, after 72 h, corresponding to yields of 63% and 62% of the theoretical, respectively.

Although the same pretreatment method was applied to both materials used in this study, SPWS 1 and SPWS 2 had quite different characteristics. This appears to be due to the different temperature control systems used for SPWS 2. Better temperature control reduced the temperature overshoot during the first few minutes of pretreatment and decreased the amount of steam admitted into the reactor, resulting in slightly milder pretreatment conditions and higher WIS.

Hydrolysis of the solid fraction of the SPWS was performed at 18.5% WIS supplemented with Cellic Ctec2 at an enzyme loading of 14 FPU/g glucan. The design of the solid-state fermentor allowed adequate mixing from the very beginning of hydrolysis and the slurry became liquefied already after 5 h allowing homogeneous samples to be taken. The final glucose and xylose concentrations were 101.7 g/L and 19.9 g/L, respectively, after 120 h, corresponding to 75% and 69% of the theoretical yield. These yields are higher than those obtained by Jorgenssen *et al. *[34], which would have been about 60% and 55% for glucose and xylose, respectively, recalculated assuming a linear correlation between cellulose and hemicellulose conversion and solid loading (WIS). The supernatant of the hydrolyzed SPWS solid fraction was used as a sugar-rich component in the feed for fermentation in Config. 2. Since the optimization of enzymatic hydrolysis was not the aim of this study, further investigations will be required to find the optimum temperature, WIS, and enzyme loading.

### Fermentation

#### Glucose-fed fermentation

Fermentation of the SPWS hydrolysate (Config. 1) or the liquid fraction of SPWS (Config. 2) was first carried out in a simplified way, using a glucose solution as the feed instead of wheat-starch hydrolysate (Config. 1_G _and Config. 2_G_). The results obtained are presented in Table 1.

**Table 1:** Results obtained from fermentation fed with glucose solution (different concentrations in Config. 1G and Config. 2_G_^a^)

Expt **Config**.	Yeast load (g/L)	Xylose consumption^b ^(%)	Xylose conc. (g/L)	Xylitol formation^c ^(%, g/L)	Glycerol conc. (g/L)	Ethanol conc. (g/L)	Ethanol yield (g/g)	Ethanol yield^d ^(%)
Config. 1_G_	5	98	0.4	19 (4.3)	6.2	53.1	0.48	93
Config. 2_G_	5	93	1.6	12 (2.6)	5.4	60.5	0.53	104

The raw material used for the experiments was SPWS 1.

^a^See Methods.

^b^Related to the total amount of available xylose.

^c^Related to consumed xylose.

^d^Corresponding to the maximum theoretical yield from available glucose and xylose.

G, glucose feed.

**Figure 1:**
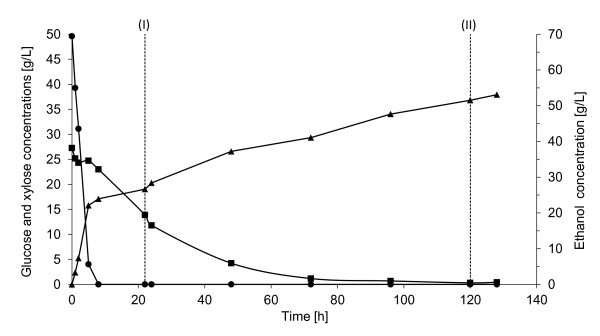
**Concentrations of glucose (circles), xylose (squares), and ethanol (triangles) during fermentation in Config. 1_G _with 5 g/L yeast**. Glucose solution was fed to the fermentor for 96 h between (I) and (II) at a constant flow rate of 2.1 mL/h. (T: 32°C, pH 5, 300 rpm).

In Config. 1_G_, fermentation was carried out on SPWS hydrolysate obtained after enzymatic hydrolysis, using the whole SPWS slurry with a WIS content of 7.5%. Figure 1 shows the concentration profiles of the substrates and the ethanol during fermentation. The glucose concentration was 50 g/L at the start of fermentation, corresponding to a glucose:xylose ratio of about 2 in the supernatant, which is very high. This sugar ratio could have a negative effect on the xylose uptake, but the glucose was consumed rapidly during the first 8 h, after which xylose uptake took place. More than 90% of the glucose was consumed in the first 5 h, resulting in a volumetric ethanol productivity of 4.4 g/L h during this time. Surprisingly, some xylose was consumed during this period, although has been claimed that the transport of xylose into the cells of genetically modified *S. cerevisiae *is inhibited by glucose [35]. When the glucose concentration fell below 5 g/L, the xylose uptake increased to 0.11 g/g cell h. Although, after 8 h there was no glucose in the medium, the xylose uptake reached 0.14 g/g cell h.

The volumetric ethanol productivity decreased from 3.0 to only 0.3 g/L h after the first 8 h, since xylose was the only available sugar in the medium. The low glucose feed rate (0.6 to 0.5 g/L h) started after 22 h (labeled I in Figure 1) and continued for 96 h. This corresponds to the glucose uptake as all the glucose was immediately consumed, that is, the glucose concentration in the medium was below the detection level. After 24 h the xylose uptake gradually declined, but almost all the xylose was consumed after 72 h, and 98% by the end of the fermentation period (120 h). These results are similar to that observed by Olofsson [21], who reported almost complete xylose consumption when glucose feed was applied during fermentation of wheat straw hydrolysate, although the initial glucose:xylose ratio in their study was one order of magnitude lower. The xylose consumption rate was lower in the present study (0.11 g xylose/g cell h *vs*. 0.15 g/g cell h), which is probably due to the initial glucose:xylose ratio. A final ethanol concentration of 53 g/L was obtained in the present study, corresponding to 93% of the theoretical yield, which is significantly higher than that obtained by Olofsson, 71% [21]. Besides ethanol, 4.3 g/L xylitol and 6.2 g/L glycerol were also formed in our experiment; mostly during the first 24 h. Small amounts of lactic acid (5 g/L) were also produced after 72 h of fermentation.

From this experiment, it can be concluded that glucose present from the beginning is consumed already in 5 h. The aim was to start feeding when most of the glucose was consumed and to provide a small amount of glucose during the fermentation. As a result the batch-fermentation phase could be shortened to only 5 h. Complete xylose consumption already after 72 h made it possible to shorten the feeding period, and lower the residence time required for fermentation.

**Figure 2:**
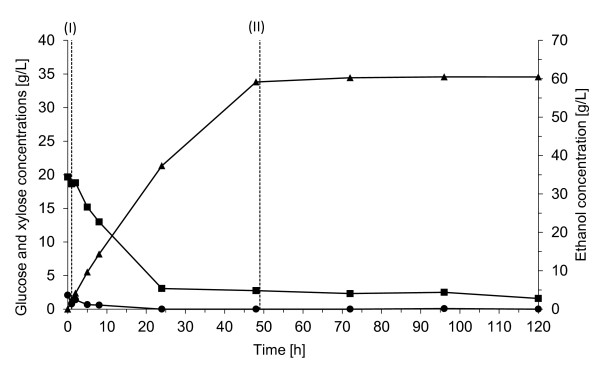
**Concentrations of glucose (circles), xylose (squares), and ethanol (triangles) during fermentation in Config. 2_G _using 5 g/L yeast**. Glucose solution was fed to the fermentor for 48 h between (I) and (II) at a constant flow rate of 4.2 mL/h. (T: 32°C, pH 5, 300 rpm).

In Config. 2_G_, lignocellulose hydrolysate following pretreatment was fermented with an initial yeast concentration of 5 g/L. Figure 2 shows the concentrations of the products and substrates during fermentation. The initial glucose concentration in the medium was very low, 2.1 g/L. Glucose solution feeding was started already after 1 h (labeled I in Figure 2). Cellulose enzymes were also added at that time to ensure the breakdown of the oligomer sugars in the hydrolysate. The flow rate of the feed was constant, but since the volume was increasing, the rate of glucose feeding changed from 2.2 g/L h to 1.8 g/L h linearly during the 48-h feeding time. This feed rate corresponds to an average of 0.44 g glucose/g cell h, and is in the same range as the glucose release rate (0.5 g glucose/g cell h, calculated by the authors) that showed the best results in the study by Olofsson [21] on SSCF with TMB3400. The xylose uptake during the first 8 h was much faster, than in Config. 1_G _(0.17 g/g cell h vs. 0.11 g/g cell h), probably due to the lower initial glucose concentration. However, xylose consumption had almost ceased after 24 h, when the xylose concentration fell below 4 g/L. At this point the glucose:xylose ratio in the medium became too high, and glucose utilization was prioritized over xylose, since they are competitively transported by the same transport protein [25]. At the end of the fermentation period (120 h) 1.5 g/L xylose remained in the broth, resulting in 93% xylose consumption. The xylose consumption in this experiment was sufficient to apply a similar feeding strategy also when the experiments were fed with real hydrolysates.

#### Wheat-starch-hydrolysate-fed fermentation

**Figure 3:**
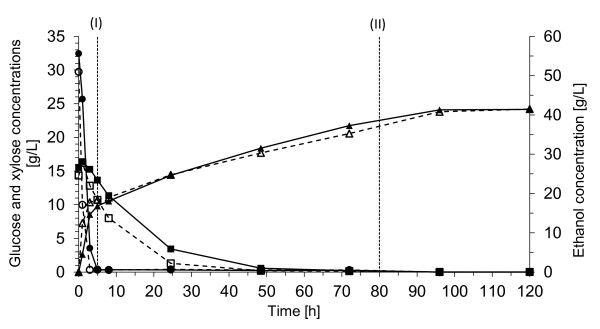
**Concentrations of glucose (circles), xylose (squares), and ethanol (triangles) during fermentation in Config. 1 using 5 g/L yeast (filled symbols) or 20 g/L yeast (open symbols)**. Wheat starch hydrolysate was fed to the fermentor for 75 h between (I) and (II) at a constant flow rate of 2.1 mL/h. (T: 32°C, pH 5, 300 rpm).

**Figure 4:**
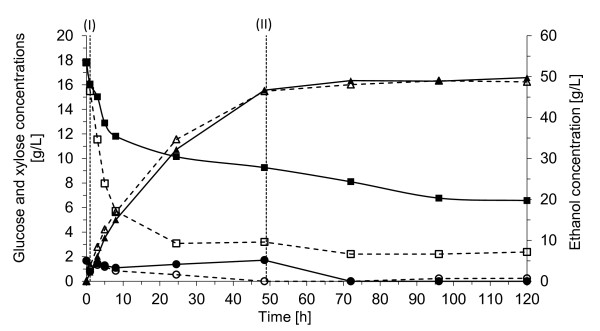
**Concentrations of glucose (circles), xylose (squares), and ethanol (triangles) during fermentation in Config. 2 with a yeast concentration of 5 g/L (filled symbols) or 20 g/L (open symbols)**. The mixed hydrolysates of SPWS hydrolysate and wheat-starch hydrolysate was fed to the reactor for 48 h between (I) and (II) at a constant flow rate of 11.2 mL/h. (T: 32°C, pH 5, 300 rpm).

Wheat-starch hydrolysate feed was used in these experiments as an alternative glucose source. The effect of increased cell mass concentration was also investigated. All the results after 120 hours' fermentation are given in Table 2. Figures 3 and 4 show the concentrations of the sugars and the ethanol during fermentation in Config. 1 and Config. 2, respectively.

**Table 2:** Results obtained after 120 h of fermentation in Config.

**Expt Config**.	Yeast load (g/L)	Xylose consumption^b ^(%)	Xylose conc. (g/L)	Xylitol formation^c ^(%, g/L)	Glycerol conc. (g/L)	Ethanol conc. (g/L)	Ethanol yield (g/g)	Ethanol yield^d ^(%)
Config. 1	5 (fresh)	100	0.0	20 (2.5)	6.9	43.1	0.49	97
Config. 1	5 (fresh)	98	0.0	20 (2.6)	5.7	43.0	0.48	93
Config. 1	5 (2w)^e^	100	0.0	21 (2.8)	5.6	41.4	0.45	88
Config. 1	20 (2w)^e^	100	0.0	13 (1.6)	6.9	41.5	0.46	91
Config. 2	5 (fresh)	76	6.5	10 (1.9)	6.6	53.3	0.44	87
Config. 2	5 (fresh)	58	11.1	9 (1.4)	5.5	52.0	0.42	83
Config. 2	5 (2w)^e^	72	6.6	9 (1.4)	5.6	49.8	0.44	87
Config. 2	20 (2w)^e^	90	2.4	13 (2.7)	7.6	49.0	0.43	84

1 or Config. 2a

^a^The raw material used for the experiments was SPWS 2.

^b^Related to the total amount of available xylose.

^c^Related to consumed xylose.

^d^Corresponding to the maximum theoretical yield from both glucose and xylose.

^e^Yeast stored in a refrigerator for two weeks (see Methods).

Config. 1, wheat-starch hydrolysate feed; Config. 2, mixed hydrolysates feed.

##### Config. 1 with wheat-starch hydrolysate feed

Wheat-starch hydrolysate feed was used in Config. 1 and was fed to the fermentors at a constant rate for 75 h after the glucose concentration had fallen to 0 g/L (Figure 3). The ethanol yield after fermentation reached an average of 95% of the theoretical based on available glucose and xylose, corresponding to a concentration of about 43 g/L ethanol, when using a yeast concentration of 5 g/L. The xylose was taken up completely; however, approximately 20% of the consumed xylose resulted in xylitol formation (Table 2). Xylitol production commenced when xylose consumption started, and it was produced until there was no xylose available in the medium; after which the xylitol concentration decreased somewhat until the end of fermentation. This phenomenon has been observed previously in defined media, suggesting that the glucose feed is insufficient to provide ATP for maintenance and glycerol production [21]. Xylitol is usually produced in *S. cerevisiae *strains that harbour the eukaryotic xylose utilization pathway (XR/XDH), due to the redox imbalance caused by the inability of the cell to provide enough NAD^+ ^for the XDH reactions [18]. The xylitol formation was in the same range as that observed in a study by Olofsson *et al. *on wheat straw hydrolysate at 30°C [21], but much lower than the yield obtained on defined medium in the same study. This is due to external electron acceptors such as furfural or hydroxyl-methyl-furfural, which are present in the lignocellulose hydrolysate, which reduce xylitol formation [17]. However, the concentration of the inhibitors was very low, and the furfural present was assimilated very quickly. It may be advantageous to apply somewhat more severe pretreatment to generate slightly more sugar degradation products, which, up to a certain concentration, have been shown to increase the ethanol yield [32,36].

##### Effect of increased yeast concentration in Config. 1

Increasing the yeast concentration to 20 g/L gave similar results regarding the ethanol yield, the ethanol concentration and xylose uptake, but the xylitol formation decreased to 12% of the consumed xylose (Table 2). The glycerol concentration increased from 5.6 g/L to 6.9 g/L, which is probably due to the increased cell concentration. The xylose was completely consumed after 48 h, thus a feeding rate 0.8 g glucose/L h is sufficient to enhance xylose consumption. A higher glucose feed rate could be applied but this may lead to a decrease in the xylose uptake. This is exactly what can be seen when the glucose feed rate was increased in the experiments in Config. 2. Whether a shorter residence time would be more beneficial, apart from the complete xylose uptake can only be determined by an economic evaluation.

##### Config. 2 with mixed hydrolysates feed

**Figure 5:**
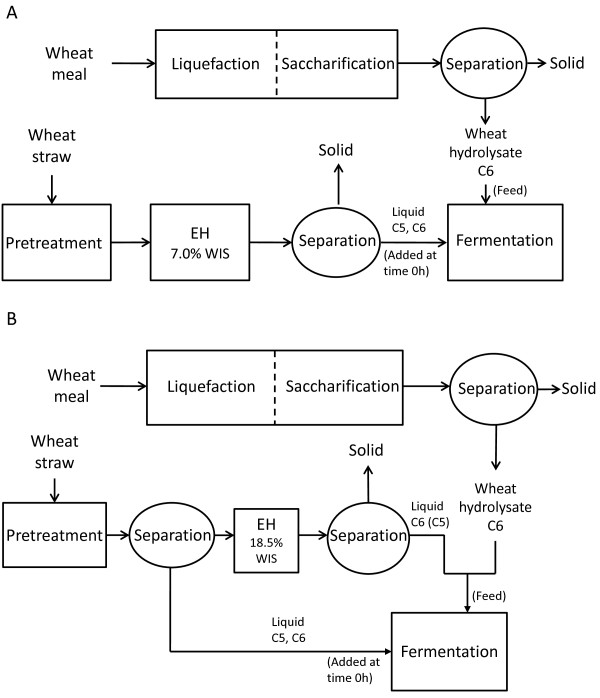
**Experimental configurations used to investigate the fermentation of steam-pretreated wheat straw**. **(A) **Config. 1, separate hydrolysis of the whole SPWS slurry and co-fermentation of glucose and xylose in the SPWS hydrolysate fed with wheat-starch hydrolysate (or with glucose solution). **(B) **Config. 2, co-fermentation of glucose and xylose in the liquid fraction of SPWS, which was fed with the mixed hydrolysates of SPWS hydrolysate and wheat-starch hydrolysate, (or with glucose solution).

Config. 2 is a possible alternative for controlled glucose feeding, by separating some of the SPWS liquid fraction from the fibre residue (without washing) before enzymatic hydrolysis with a high WIS loading. This would produce a glucose-rich supernatant, which could be used as feed in the fermentation. In the present study the wheat-starch hydrolysate was also mixed with the feed in order to investigate the integrated process. The liquid fraction of SPWS was used in the fermentation and then fed with the mixed hydrolysates for 48 h (Figures 4 and 5B). One of the drawbacks of this configuration is that a considerable amount of xylose is present in the feed, which in this case led to remarkably decreased xylose consumption during the second 24-h period of feeding (Figure 4). The ethanol yield with an initial yeast concentration of 5 g/L was between 83% and 87% (Table 2), corresponding to an ethanol concentration of about 50 g/L. The xylose consumption varied between 58% and 76% with very low xylitol formation in relation to the xylose uptake (< 10%), corresponding to only 1.4 g/L xylitol after 120 h of fermentation. The glycerol concentration was 5.6 g/L during the last hour. These results are in the same range as those reported for barley straw hydrolysate [30], although the best xylose uptake was observed with a much lower glucose feed rate of 0.21 g/L h.

##### Increased yeast concentration in Config. 2

High, yeast concentration (20 g/L) was also investigated, but this did not improve the ethanol yield, despite the fact that the xylose uptake increased to 90%. However, the xylitol and the glycerol concentrations increased to 2.7 g/L (13% of the consumed xylose) and 7.6 g/L, respectively.

##### Comparison of the two configurations

Despite the high initial feed rate in Config. 2, 4.4 g/L h, corresponding to 0.88 g glucose/g cell h, the glucose:xylose ratio was still low enough to result in a faster xylose uptake than that obtained in Config. 1 with 5 g/L cell mass, with a much higher initial glucose concentration. After 8 h as the xylose concentration decreased; however, to maintain the glucose:xylose ratio the feed rate should also have been adjusted, thought it would have been rather difficult as there was also xylose present in the feed. Accumulation of glucose, which was observed (Figure 4), should be avoided, indicating that a lower glucose feed rate should be applied. Nevertheless, the xylose was completely assimilated in Config. 1, which indicates that the initial glucose:xylose ratio has an influence on the initial xylose uptake, but not on the final uptake. As a higher ethanol yield was reached Config. 1 is a more advantageous process configuration in case of co-fermentation of xylose and glucose with the *S. cerevisiae *TMB3400. It is also less demanding from a technical point of view.

## Conclusions

Complete xylose utilization could be achieved with TMB3400 co-fermentation of glucose and xylose in SPWS hydrolysate when the glucose-rich wheat-starch hydrolysate was fed to the fermentor. A high ethanol concentration, up to 5% (wt/wt), and a high ethanol yield, above 0.42 g/g, were achieved with both configurations (Config. 1 and 2), based on the utilization of both glucose and xylose; however, the yields and the xylose consumption were higher in Config. 1. Although increased cell concentration did not result in improved ethanol yield in fermentation, an improved xylose uptake rate was observed due to the increased fermentation capacity. Integration of the first-generation process into the second-generation (2 G) process also increases the ethanol concentration, which will result in a decrease in the cost of distillation, thus improving process economy. In this way, introduction of the 2 G bioethanol production may be considerably facilitated.

## Methods

Two configurations for SHCF of glucose and xylose were used to investigate the effect of controlled wheat-starch hydrolysate feed on the xylose uptake. Both are illustrated in Figure 5. The first configuration (Figure 5A, denoted Config. 1) was used for assessment of glucose and xylose co-fermentation in the SPWS hydrolysate. The whole slurry of SPWS 1 or 2 was diluted to 7.5% or 7.0% water insoluble solids (WIS) prior to enzymatic hydrolysis, which was followed by separation of SPWS hydrolysate and the solid fraction. SPWS hydrolysate was then subjected to fermentation. The supernatant from the liquefied and saccharified wheat meal (wheat-starch hydrolysate) was used as feed to the fermentor, commencing when the glucose present in the SPWS hydrolysate had been consumed. The extensive amount of C6 sugars present in the SPWS hydrolysate, resulting in a high glucose:xylose ratio, may have a negative effect on the xylose fermentation.

In order to investigate the glucose and xylose consumption and to obtain information about the feeding strategy, the method was simplified so that wheat-starch hydrolysate was replaced by glucose solution at a glucose concentration corresponding to that of the hydrolysate. Using glucose solution in the first trials (Config. 1_G _and Config. 2_G_) was also motivated by the simpler preparation procedure and the more exact dosing of glucose.

Batch fermentation in Config. 1_G _was run for 24 h, and the glucose solution was then fed into the fermentor for 96 h. Based on the results of this experiment, it was decided to feed wheat-starch hydrolysate in Config. 1 for 75 h after 5 h of batch fermentation.

Another configuration, Config. 2 (Figure 5B), was used where the SPWS slurry was pressed to 18.5% dry matter (DM), enzymatically hydrolyzed and then separated into SPWS hydrolysate and a solid fraction. SPWS hydrolysate was mixed with the wheat-starch hydrolysate (mixed hydrolysates) and used as feed for fermentation. In the fermentation step the liquid fraction of SPWS 2 slurry was fermented for 1 h in order to ferment the glucose already present and then fed with glucose solution (Config. 2_G_) or with the mixed hydrolysates (Config. 2) for 48 h. As opposed to Config. 1, a small amount of C6 and a large amount of C5 sugars were present in the broth in Config. 2 from the beginning of the fermentation. More C6 sugars were present in the feed in this case, which could be advantageous for the xylose fermenting *S. cerevisiae *TMB3400; however, xylose in the feed may have a counterproductive effect. Both configurations were investigated in order to assess fermentation of xylose.

### Raw materials and pretreatment

Two batches of wheat straw (Straw 1 and Straw 2) were used in the study. Both were kindly provided by Johan Håkansson Lantbruksprodukter (Lunnarp, Sweden). Straw 1 and Straw 2 consisted of about 93% and 92% DM, respectively. Their compositions are given in Table 3.

**Table 3:** Composition of the two batches of wheat straw, expressed as a percentage of dry matter

	Straw 1 (%)	Straw 2 (%)
Glucan	38.6	34.2
Xylan	25.8	25.2
Arabinan	3.9	3.3
Lignin	20.4	21.9
Ash	4.4	4.1

The straw was milled with a knife-mill and then sieved to obtain particles in the range of 2 to 10 mm. The milled straw was stored in plastic bags at room temperature prior to impregnation. Impregnation was performed with 0.2% sulphuric-acid solution at a liquid:dry matter ratio of 20 for 1 h, and the straw was then pressed to an average DM content of 50% and stored in sealed plastic buckets overnight until pretreated. The impregnated straw was pretreated in a steam pretreatment unit, described previously [37], at 190°C for 10 min, as it was previously optimized by Linde *et al. *[38]. The steam pretreated wheat straw (SPWS) slurry was thoroughly mixed and stored at 4°C. Total DM and WIS contents were determined from three representative samples. Soluble sugars, degradation products, and total soluble sugars after acid hydrolysis were determined from the liquid fraction, according to the standardized analytical procedures of the National Renewable Energy Laboratory (NREL) [39]. The solid fraction of SPWS was extensively washed with hot water in order to remove all soluble substances, dried, and milled before compositional analysis. The structural carbohydrates and lignin content of this solid residue were determined according to the NREL method [40]. The composition of the SPWS from the two batches (SPWS 1 and 2) is given in Table 4. Straw 2 was pretreated after modification of the control system providing improved temperature control. This might explain the increased WIS content in the batch of SPWS 2. SPWS 1 was used for the experiments run with glucose feed only (Config. 1_G _and Config. 2_G_), while the SPWS 2 material was used for all the trials run with the real wheat-starch hydrolysate feed (Config. 1 and Config. 2).

**Table 4:** Composition of the two batches of steam-pretreated wheat straw (SPWS)

			SPWS 1	SPWS 2
Total solids (%)			12.3	18.7
WIS content (%)			8.2	13.5
Solid fraction (% of WIS)		Glucan	62.2	58.6
		Xylan	5.8	6.3
		Arabinan	0.6	0.4
		Lignin	23.5	27.7
		Ash	3.4	3.0
Liquid fraction (g/L)	Sugars^a^	Glucose	5.0	9.8
		Xylose	30.4	43.7
		Arabinose	3.9	5.8
	By-products	Furfural	2.0	1.7
		HMF	0.1	0.1
		Acetic acid	2.9	2.4

^a^Total sugars containing both monomer and oligomer sugars.

Wheat meal (dry milled grain) with an average particle size of 2.5 mm was kindly provided by Sileco (Halland, Sweden) and stored in plastic bags at 5°C prior to use. Wheat meal processing, liquefaction, and subsequent saccharification are described below in the section on enzymatic hydrolysis.

### Cell cultivation

#### Inoculum

The inoculum culture of *Saccharomyces cerevisiae *TMB3400 was prepared on xylose-supplemented YPD plates (10 g/L yeast extract, 20 g/L peptone, 20 g/L glucose, 20 g/L xylose, and 15 g/L agar). The cells were added to a 300-mL Erlenmeyer flask with 70 mL aqueous solution containing 23.8 g/L glucose, 23.8 g/L xylose, 10.8 g/L (NH_4_)_2_SO_4_, 5.0 g/L KH_2_PO_4_, and 1.1 g/L MgSO_4 _7H_2_O. 14.4 mL/L trace metal solution and 1.4 mL/L vitamin solution was prepared according to Taherzadeh *et al. *[41] and also added to the solution. The pH was adjusted to pH 5 with 0.25 M NaOH. The Erlenmeyer flask was sealed with a cotton plug and incubated on a rotary shaker at 30°C for 24 h at 180 rpm.

#### Batch cultivation

Batch cultivation was performed in a 2-L LABFORS fermentor (Infors AG, Bottmingen, Switzerland) with a working volume of 0.5 L. Cultivation was started by adding 70 mL inoculum to a medium containing 20.0 g/L glucose, 22.5 g/L (NH_4_)_2_SO_4_, 10.5 g/L KH_2_PO_4_, 2.2 g/L MgSO_4 _7H_2_O, 60.0 mL/L trace metal solution, and 6.0 mL/L vitamin solution. The pH was adjusted to 5 with 2.5 M NaOH. The stirrer speed was set to 700 rpm and the aeration rate was 1 vvm, corresponding to an average of 0.5 L/min. The dissolved oxygen concentration was continuously measured with an oxygen sensor. Fed-batch cultivation was started when the oxygen concentration showed a rapid increase, indicating that all the sugar and the ethanol had been consumed.

#### Fed-batch cultivation

Batch cultivation was followed by a 24-h fed-batch phase, since short-term adaptation of the cells used for fermentation of lignocellulosic hydrolysate has been shown to improve fermentation performance [42]. Fed-batch cultivation was performed by the continuous addition of 847 mL SPWS liquid supplemented with glucose and salt solution to a total volume of 1 L, resulting in a final volume of 1.5 L. The glucose concentration in the SPWS liquid solution was adjusted to 70.0 g/L. Salts were added to the solution to concentrations of 11.3 g/L(NH_4_)_2_SO_4_, 5.3 g/L KH_2_PO_4_, and 1.1 g/L MgSO_4 _7H_2_O. The final concentration of diluted SPWS liquid was equivalent to that obtained when the slurry from pretreatment had been diluted to 7.5% WIS. The pH of the hydrolysate was adjusted to pH 5 with solid NaOH. The SPWS liquid solution was added to the fermentor at a constant rate of 125 mL/h for 24 h. The pH was continuously adjusted to pH 5 with 2.5 M NaOH. The stirrer speed was increased to 900 rpm and the rate of aeration to 1.5 vvm. When all the sugar and the ethanol produced had been consumed, cultivation was stopped and the culture broth was centrifuged at 4,000 rpm for 10 min. The supernatant was discarded and the DM of the harvested cells was determined before use in fermentation. The cells used for starch-hydrolysate-fed fermentation with 20 g/L cell concentration were stored in a refrigerator for two weeks (it has previously been found that the performance of this yeast does not change when stored in a refrigerator for up to a month, data not shown). However, reference fermentation experiments supplemented with the 5 g/L stored yeast were run in parallel.

### Enzymatic hydrolysis

#### Config. 1

Enzymatic hydrolysis of the whole SPWS slurry in Config. 1_G _was performed in a 20-L bio-lab reactor (Bioengineering AG, Wald, Switzerland) with a total working weight of 10 kg. The WIS concentration of the SPWS 1 material was adjusted to 7.5% and hydrolysis was carried out at 40°C, pH 4.8 for 120 h, at 850 rpm. Cellic CTec cellulase (95 FPU/g, 590 β-glucosidase IU/g, 712 xylanase IU/g, and 63 β-xylanase IU/g) and Cellic HTec xylanase (7998 xylanase IU/g and 99 β-xylanase IU/g) enzyme preparations were added at a ratio of 10:1 based on the glucan content of the WIS in the steam pretreated material (20 FPU/g glucan). Both enzyme preparations were kind gifts from Novozymes A/S (Bagsværd, Denmark).

Enzymatic hydrolysis in the starch-hydrolysate-fed experiments (Config. 1) was performed using SPWS 2 at a WIS content of 7.0%, using the newly released cellulase enzyme, Cellic CTec2 (Novozymes A/S) cellulase (128 FPU/mL, 4465 β-glucosidase IU/mL) at an enzyme loading of 14 FPU/g glucan. The reason for the different enzyme loading is that the enzyme preparation has a high concentration of glucose (280 g/L), which we were not aware of when the experiments were designed. The FPU activity determined before the experiments was actually 24% higher than the actual value corrected for the glucose content. The parameters of the experiments were therefore recalculated afterwards, resulting in an FPA dosage of 14 FPU/g glucan instead of the intended 20 FPU/g. The liquid fraction (SPWS hydrolysate) after this step was separated from the solid fraction with a press-filter and then subjected to fermentation.

#### Config. 2

Enzymatic hydrolysis of the solid fraction of SPWS 2 slurry with 18.5% WIS was performed in two batches, 3.6 and 3.1 kg, in a 12-L solid-state fermentor (Terrafors, Infors AG). The temperature in the fermentor was maintained at 40°C with the help of an external water bath. The pH was set to 5 at the beginning of hydrolysis and when the slurry became more liquefied the pH was readjusted with 50% NaOH in conjunction with sampling. Cellic CTec2 enzyme preparation was used at a dosage of 14 FPU/g glucan. After hydrolysis, the liquid fraction (SPWS hydrolysate) was separated from the solid fraction. The SPWS hydrolysate was mixed with the wheat-starch hydrolysate (mixed hydrolysates) before being fed to the fermentation vessel.

#### Wheat hydrolysate

In order to produce wheat-starch hydrolysate, wheat meal was enzymatically treated using two-step hydrolysis, consisting of liquefaction and saccharification. The hydrolysis of 1.2 or 5 kg batches was performed using a 2- or 10-L evaporator (Büchi Labortechnik AG, Flawil, Switzerland). In the first step, wheat meal slurry (35% DM) was liquefied by thermostable α-amylases (Termamyl SC; Novozymes A/S, Bagsværd, Denmark) at 85°C, pH 5.5 for 3 h. Termamyl was added at a dosage of 0.5 g enzyme/kg DM meal. In the following step, amyloglucosidase (Spirizyme Fuel; Novozymes A/S) was loaded at a level of 1 mL/kg DM after the pH had been adjusted to 4.2 and the temperature reduced to 60°C. Saccharification was performed at 60°C for 24 h to ensure complete breakdown of the oligomers into monomer sugars. The hydrolysis broth was centrifuged twice, first at 4,000 rpm, for 10 min in 500-mL flasks. The supernatant was then transferred to 50-mL centrifuge tubes and centrifuged at 4,500 rpm for 10 min. The supernatant was saved and frozen prior to further use. The glucose concentration in the starch hydrolysate was determined to be between 316 and 340 g/L in different batches.

### Ethanol fermentation

The experimental set-up for each fermentation performed, working volumes, feed volumes, and the total amount of sugar added in the different fermentation experiments are presented in Table 5. The sugar concentrations in the different fermentation experiments varied due to the different amounts of sugar after enzymatic hydrolysis, but the yields were always calculated based on the actual amount of sugar added.

**Table 5:** Experimental set-up and the amount of sugars^a ^added at 0 h and with the feed^b ^in the various fermentation experiments

Feed	Expt Config	Yeast load (g/L)	Feeding start; stop (h)	G_feed rate _(g/h)	V_final _(L)	V_feed _		Glucose_added at 0h_ (g)	Xylose_added at 0h_ (g)	Glucose_feed_		Xylose_feed_ (g)
						**SPWS hydr. (mL)**	**Starch hydr. (mL)**			**SPWS hydr. (g)**	**Starch hydr. (g)**	

Glucose (_G_)	Config. 1_G_	5	22; 120	0.6	1.2	180^e^		49.6	27.3	56.3^f^		-
	Config. 2_G_	5	1; 49	2.2	1.2	180^e^		4.4	27.2	104.7^f^		-
Wheat-	Config. 1	5	5; 80	0.8	1.2		180^d^	31.7	15.4	-	57.0	-
starch	Config. 1	5	5; 80	0.8	1.2		180^d^	31.7	15.4	-	60.6	-
hydrolysate	Config. 1	5^c^	5; 80	0.7	1.0		150^d^	27.6	13.2	-	50.5	-
	Config. 1	20	5; 80	0.7	1.0		150^d^	25.3	12.2	-	50.5	-
	Config. 2	5	1; 49	2.3	1.2	492	180^d^	4.8	21.5	50.0	57.0	9.8
	Config. 2	5	1; 49	2.3	1.2	492	180^d^	4.8	21.5	50.0	60.6	9.8
	Config. 2	5^c^	1; 49	1.8	1.0	367	150^d^	3.4	15.2	35.2	50.5	7.6
	Config. 2	20	1; 49	1.8	1.0	367	150^d^	3.4	15.2	35.2	50.5	7.6

^a^Glucose and xylose

^b^Glucose, SPWS hydrolysate, and wheat-starch hydrolysate.

^c^Reference for the 20 g/L run with the yeast stored in the refrigerator for two weeks.

^d^Wheat-starch hydrolysate in the feed mixture (17% of V_final_).

^e^Volume of glucose solution feed

^f^Glucose added with the glucose solution feed.

G, glucose.

#### Fermentation with glucose feed

Glucose-fed fermentations were performed in 2-L LABFORS bioreactors (Infors AG) with a working volume of 1.2 L. The SPWS hydrolysate obtained from the enzymatic hydrolysis of material containing 7.5% WIS in Config. 1_G_, or the liquid fraction of SPWS (diluted with water to a concentration equivalent to that which would have been obtained if the slurry from pretreatment had been diluted to 7.5% WIS) in Config. 2_G _was added to the fermentor and autoclaved at 120°C for 20 min. Nutrients were dissolved separately in water at final concentrations of 1 g/l (NH_4_)_2_HPO_4_, 0.05 g/L MgSO_4 _7H_2_O, and 2 g/L yeast extract, sterilized, and added to the bioreactor before inoculation. The nutrient concentration was doubled when the cell concentration of 20 g/L was used. The pH of the hydrolysates was set to 5 using solid NaOH before autoclaving and then controlled with 10% NaOH solution during fermentation. Fermentation was carried out at 300 rpm and 32°C for 120 h.

In Config. 1_G _fermentation was allowed to continue for another 8 h after feeding had ceased (120 h) before it was stopped, so that all the glucose could be consumed. Nitrogen gas was flushed through the bioreactors before fermentation to ensure an anaerobic environment. Yeast cell suspensions at a dry cell concentration of 5 g/L were added to the fermentors. Then 180 mL glucose solution was fed to each fermentor (Table 5). The solutions had different concentrations in each configuration, such that the amount of glucose added would be the same as when using wheat-starch hydrolysate.

After 22 h, a glucose solution at a concentration of 323 g/L was fed to the fermentation vessel of Config. 1_G _for another 96 h at a constant flow rate. The glucose addition of 0.6 g/h decreased from 0.6 to 0.5 g/L h linearly as the volume increased due to the feed. The solution prepared had approximately the same glucose concentration as the wheat hydrolysate.

In Config. 2_G _the feed had a higher concentration of glucose (600 g/L). It was also added at a constant flow rate but for only 48 h, starting after 1 h. The extra glucose added corresponded to the amount of glucose theoretically available in the form of glucan in the WIS fraction, which would have been hydrolyzed separately. Due to the volume increase, the rate of glucose addition changed linearly from 2.2 to 1.8 g/L h during the 48 h. One hour after starting fermentation Cellic CTec and Cellic HTec mixture (10:1) was added at a dosage of 5 FPU/g (theoretical) WIS to ensure the breakdown of the oligomers present in the hydrolysate.

#### Fermentation with wheat-starch hydrolysate feed

The experiments were performed in the 2 L Labfors fermentors for 120 h using a working volume of 1.2 or 1.0 L (Table 5). In Config. 1 SPWS hydrolysate, after the 7.5% enzymatic hydrolysis or the liquid fraction of SPWS in Config. 2 was added to the fermentor and autoclaved at 120°C for 20 min. The fermentations were initiated by adding cell suspension, which would result in a concentration of 5 g/L in the final volume. The fermentation parameters, such as pH, temperature, stirrer speed, and the nutrient concentration were the same as in the fermentations with glucose feed. The fermentation of Config. 1 was then fed with wheat-starch hydrolysate and Config. 2 with the mixed hydrolysates described above. An increased cell concentration, 20 g/L, was also used in another set of experiments, to allow for comparison with these experiments which were run with yeast that had been stored in a refrigerator for two weeks.

### Sampling and analysis

Samples were withdrawn frequently after the first hour. The concentrations at 0 h were calculated from the known concentrations of the input materials. Samples taken from enzymatic hydrolysis and fermentation were centrifuged in 10-mL centrifuge tubes at 4,000 rpm for 5 min (Labofuge 200; Thermo elektron LED GmbH, Osterode, Germany). The supernatant was filtered using 0.2-μm filters, and the filtered samples were stored at -20°C prior to analysis.

Hydrolysate samples after pretreatment, samples from the NREL fiber and total soluble sugars analysis, and samples withdrawn from enzymatic hydrolysis and fermentation were analyzed using a high-performance liquid chromatograph equipped with a refractive index detector (both from Shimadzu, Kyoto, Japan). Cellobiose, glucose, xylose, galactose, arabinose, and xylitol were separated on an Aminex HPX-87P column (Bio-Rad Laboratories, Hercules, CA, USA) at 85°C with Millipore water at a flow rate of 0.6 mL/min. Ethanol, glycerol, lactic acid, acetic acid, HMF, and furfural were separated on an Aminex HPX-87H column (Bio-Rad Laboratories) at 50°C, with 5 mM H_2_SO_4 _at a flow rate of 0.5 mL/min. All samples were neutralized with CaCO_3_(s) and filtered through a 0.2-μm filter before analysis.

### Yield calculations

The sugar yield in enzymatic hydrolysis was calculated on the basis of the total available sugars in the liquid and the solid fraction of SPWS. The theoretical amount of glucose released during hydrolysis is 1.11 times the amount of glucan and 1.13 times the amount of xylan in the solid material (due to the addition of water in hydrolysis). The ethanol yield was calculated based on the total amount of fermentable sugars added to the fermentation stage, that is, the sum of available glucose and/or xylose in the liquid fraction of SPWS (including monomers and oligomers) and in the feed. Based on a maximum ethanol yield of 0.51 g/g (yield from hexoses and xylose), the percentage of theoretically available (Y*_E/S_, %) was calculated as Y*_E/S _= (Y_E/S_/0.51)*100, where Y_E/S _is the ethanol yield from the sugar substrates.

## Abbreviations

DM: dry matter; FPA: filter paper activity; FPU: filter paper unit; NREL: national renewable energy laboratory; SHCF: separate hydrolysis and co-fermentation; SHF: separate hydrolysis and fermentation; SSCF: simultaneous saccharification and co-fermentation; SSF: simultaneous saccharification and fermentation; SPWS: steam-pretreated wheat straw; WIS: water insoluble solids; YPD: yeast extract peptone dextrose; XDH: xylitol dehydrogenase; XI: xylose isomerase; XK: xylulokinase; XR: xylose reductase.

## Competing interests

The authors declare that they have no competing interests.

## Authors' contributions

BE, MG, and GZ designed and coordinated the study. BE and BF carried out the experiments and analyzed the results. BE prepared the manuscript. MG and GZ reviewed the manuscript. All authors read and approved the final manuscript.
